# "I was just concerned about getting pregnant”: Attitudes toward pregnancy and contraceptive use among adolescent girls and young women in Thika, Kenya

**DOI:** 10.1186/s12884-023-05802-3

**Published:** 2023-07-04

**Authors:** Afkera Kesete Daniel, Edinah Casmir, Lynda Oluoch, Murugi Micheni, Catherine Kiptinness, Anna Wald, Nelly Rwamba Mugo, Alison C. Roxby, Kenneth Ngure

**Affiliations:** 1 Johns Hopkins University, Baltimore, MD USA; 2 Center for Clinical Research, Kenya Medical Research Institute, Nairobi, Kenya; 3 Partners in Health Research and Development, Thika, Kenya; 4 National Syndemic Disease Control Council, Nairobi, Kenya; 5 University of Washington, Seattle, WA USA; 6 Vaccine and Infectious Disease Division, Fred Hutchinson Cancer Research Center, Seattle, WA USA; 7 School of Public Health, Jomo Kenyatta University of Agriculture and Technology, Box 19704-00202, Nairobi, Kenya

**Keywords:** Pregnancy, Contraceptive use, Pregnancy and contraceptive attitudes, Adolescent girls and young women, Kenya

## Abstract

**Background:**

Adolescent girls and young women (AGYW) have a high incidence of unplanned pregnancies, especially in low-resource settings. AGYW assess the overlapping risks of pregnancy, contraception, and STIs as they navigate relationships. Few studies have examined how AGYW consider the comparative risks of their decisions around sexual and reproductive health in this context or how risk perception influences contraceptive use.

**Methods:**

Twenty in-depth interviews (IDIs) and 5 focus group discussions (FGDs) were conducted with a subset of sexually active AGYW enrolled in the Girls Health Study (GHS), a longitudinal cohort study in Thika, Kenya, assessing HSV-2 incidence in a cohort of AGYW aged 16–20. Interview questions were focused on perspectives and decision-making around sexual and reproductive health. Interviews were conducted in both English and Kiswahili, transcribed, and coded using inductive and deductive approaches to identify emerging themes.

**Results:**

Misconceptions about long-acting reversible contraceptives (LARCs), injectables, and daily oral contraceptive pills strongly disincentivized their use among AGYW. Participants described pregnancy as undesirable, and AGYW reported prioritizing contraceptive methods that were effective and reliable in pregnancy prevention, even if not effective in preventing STI/HIV infection. Participants reported that AGYW relied heavily on emergency contraceptive (EC) pills for pregnancy prevention.

**Conclusions:**

Though the goal of avoiding unintended pregnancy was common, this did not suffice to motivate the uptake of long-term contraceptives among AGYWs. Given the convenience, cost-effectiveness, and lower perceived risk of side effects, EC pills were more likely to be accepted as a form of contraception. Understanding the reasons for AGYW’s acceptance of certain contraceptive methods over others can help future interventions better target communication and counseling about contraception and influence key drivers of AGYW behavior and decision-making around sexual and reproductive health.

## Introduction

Adolescent girls and young women (AGYW) in low and middle-income countries (LMICs) are at notably high risk of adverse sexual and reproductive health outcomes [1]. AGYW face significant barriers to pregnancy planning and STI/HIV prevention, including lack of knowledge about contraceptives, unaffordability, limited accessibility, high incidence of sexual violence and community stigma [2–4]. Around 1.6 million adolescents are living with HIV worldwide, with AGYW accounting for 79% of new infections among those aged 10–19 [5]. Globally, complications secondary to pregnancy, childbirth and unsafe abortions are among the top causes of death among AGYW aged 15 to 19 [4, 6, 7].

Early pregnancy puts adolescent mothers at higher risk of complications such as pre-eclampsia, endometritis, hemorrhage, and systemic infection compared to older women [8]. Furthermore, among adolescent girls aged 15–19, it is estimated that almost half of early pregnancies are unintended [9].Unintended pregnancy during adolescence is also associated with an array of lasting negative social and economic consequences, compromising adolescents’ future educational and economic opportunities [10]. Adolescents with unplanned pregnancies are more likely to discontinue schooling, leading to subsequent lower educational attainment and decreased social opportunities, including reduced lifetime earnings [11].

Although consistent contraception use seems strongly associated with the desire among AGYW to avoid pregnancy, particularly prior to completing educational goals or before marriage, contraceptive uptake among AGYW in LMICs remains low [9, 12, 13]. Many factors influence AGYW’s choices around pregnancy and contraceptive use, including individual opinions, social norms and living circumstances, misconceptions about contraceptives, and stigma among communities and healthcare providers [3, 4, 9]. Research has also shown that use of long-acting reversible contraceptives (LARCs) is particularly low among AGYW, while use of emergency contraception (EC) pills has been emerging as a more popular and accessible option among this group [14–16].

It is estimated that realizing the unmet need for contraception among AGYW would reduce unintended pregnancies among adolescents by 6 million annually, leading to 2.1 million fewer unplanned births, 3.2 million fewer abortions, and 5600 fewer maternal deaths [3, 9]. Public health interventions aimed at improving the reproductive health of AGYW often target barriers to accessing contraceptives and preventing sexually transmitted infections, including social structures and policies, and the agency and empowerment of adolescents themselves [17]. However, intervention strategies have largely failed to address the broader contexts that surround adolescent behaviors and outcomes. Though studies have demonstrated challenges and potential paths to reducing early and unplanned pregnancy among AGYWs, few have looked closely at these barriers and interventions in a cohort of young women followed from the initiation of sexual activity and into adulthood. We conducted a qualitative study to demonstrate patterns of decision-making around contraception choice among AGYW at the time of their early sexual experiences, with the aim of better understanding ways to increase uptake of contraceptives around the time of first intercourse.

## Methods

### Study design, setting and population

This qualitative study was nested within the Girls Health Study (GHS), a longitudinal cohort study that enrolled and followed adolescent girls in Thika, Kenya from 2014–2020 [18, 19]. As study participants, AGYW had no-cost access to reproductive health education, contraception and HIV counseling in a youth friendly environment. The cohort was designed to span the critical years before and after AGYW became sexually active.

For our qualitative research, we recruited girls from within this cohort, which provided the unique opportunity to examine decision-making around contraceptive use for a group with known access to high-quality youth-friendly reproductive health care services. Participants were recruited through community mobilization strategies, including consultations with community advisory groups and outreach to local colleges, schools, and churches. Our outreach team also provided culturally sensitive and community leader-approved education on sexually transmitted infections and reproductive health to girls and parents/guardians in the community. Adolescent girls were eligible for participation in the study if they were between 16–20 years old, had self-reported having had no more than one prior sexual partner, were willing to undergo quarterly genital examination and remain in follow up for 3 years. Participants were followed quarterly with blood tests for HSV-2 and HIV, genital exams with swabs for STIs (including herpes simplex virus 2, chlamydia, gonorrhea, and trichomoniasis), and sexual behavior questionnaires.

In-depth interviews (IDIs) and focus group discussions (FGDs) were conducted with a purposively selected subset of AGYW enrolled in GHS who self-reported their first sexual intercourse while enrolled in the study. These interviews were conducted at the AGYW earliest convenience after self-reported sexual initiation. The FGDs were comprised of 5–8 AGYW aged 16–18 years and 19–20 years who were willing to participate in group discussion. Written, informed consent was obtained from all study participants aged 18 and above; for participants’ age 18 years old or less, parental consent was sought in addition to individual assent. Ethical approval for the study was granted by the Kenya Medical Research Institute’s Scientific and Ethics Review Unit (SERU) and the University of Washington Institutional Review Board. Each AGYW provided individual written assent (< 18 years) or consent (> 18 years).

### Data collection

All IDIs and FGDs were conducted at the study site by experienced social scientists, in English or Kiswahili, depending on participants’ preferences. All interviewers participated in a 3-day protocol specific training which included a review of study objectives, qualitative research methods, IDI and FGD interview guides, and qualitative interview techniques. The semi-structured interview guide focused on risk perception around HIV/STIs and pregnancy as well as knowledge, attitudes, access and use of condoms and contraception by AGYW. The guide also explored factors influencing initiation of sexual activity, risk considerations, and motivations for sexual engagement. Specific probes in the interview guide further examined relationships with partners, sources of information on reproductive health and barriers to accessing contraception and sexual health services. Specifically, some of the questions in the interview guide included: (1) What do young women do to reduce the risk of pregnancy? (2) What forms of pregnancy protection are you aware of? (3) Could you please describe your pregnancy risk during your first sexual encounter? (4) Could you please share the methods you used to reduce pregnancy risk? (5) How was the decision to use or not to use the method made? (6) How easy was it to discuss protection use with your partner? (7) Could you please describe any challenges in obtaining contraception and how you overcame them?

Interviews lasted between 30–60 min and were translated if needed and transcribed from original recordings. Transcripts were imported and managed using Dedoose software (Socio-cultural Research Consultants, Manhattan Beach, CA USA).

### Data analysis

Alongside the interview team, three investigators (AKD, EC, and KN) developed a thematic coding framework based on topics covered in the interview guide. Interviews were coded using inductive and deductive approaches to identify emergent themes. Intercoder reliability was established by coding a random selection of transcripts for review and comparison by AKD, EC and KN. All interviews were then double-coded by two investigators (AKD and EC) using the thematic framework. Using the directed content analysis approach [20], fine codes were then generated for sub-themes that emerged from review of the first set of coded excerpts. Analytic reports were subsequently generated for interview domains related to pregnancy and contraception.

## Results

We conducted 20 IDIs and 5 FGDs. The mean age of participants at study enrollment, prior to first sexual intercourse, was 19 years old for IDIs and 18 years old for FGDs (interquartile range [IQR] 18–19; 17–19, respectively). At the time of the interview, after initial sexual experience was reported, the mean age of participants was 20 years old for both IDIs and FGDs (IQR 19–21 for both). All participants had completed at least primary school education, with 60% reporting that they were enrolled in college or university-level studies. The mean years of education for all participants was 12 (IQR 11–13).

Three primary themes around AGYW’s use of contraceptives emerged from our analysis of interview data: (1) AGYW perceived pregnancy to be undesirable, although this did not necessarily translate to increased uptake of contraception, (2) misconceptions about contraceptives led to avoidance of hormonal contraceptives, especially long-acting reversible contraceptives, and (3) participants reported preference for emergency contraceptive (EC) pills as contraception, both personally and among their peers.

### Theme 1: AGYW describe pregnancy as an undesired present outcome

As AGYW in our study shared reflections on their early sexual experiences, the risk of unintended pregnancy was consistently highlighted as their greatest concern. This risk perception was rooted in a desire to avoid negative community perceptions and fear of stunting life and educational opportunities. One participant noted in an FGD that:

“*…a baby comes with responsibilities and changes the direction of your whole life. You stop schooling. What will happen if my family knows I am pregnant and I am not yet through with school?” (FGD 4)*

For this participant, pregnancy would likely deter her from continuing schooling and damage her relationships with family members. A number of participants also expressed worry that pregnancy might lead to rejection and abandonment by their partners and, thus, the responsibility of having to care for a child without additional support.

*“Some have fear of their boyfriends denying being responsible for the pregnancy. Like when you tell a partner that the pregnancy is his, he will deny it because you don’t have evidence.” (FGD 5)*

Another participant noted that she feared being perceived negatively by peers, recalling the experience of a classmate who became pregnant:

*“I remember one day we were studying for exams, and one lady was pregnant and her friend said ‘Ngai [God]! She is pregnant’. Like if [this were] me I cannot imagine friends talking about me like that, no no…let me just stay away from pregnancy.” (FGD 2)*

Participants also expressed repeatedly that AGYW perceived pregnancy as less acceptable than the risk of contracting HIV or another sexually transmitted infection. One participant noted that *“most [girls] fear getting pregnant, they don’t usually think of the diseases [HIV]” (IDI 09)*. Another participant, describing her own experience, expressed: *‘I was not concerned with that [HIV], I was just concerned about getting pregnant” (IDI 10)*. When asked in an FGD to expound upon this discrepancy, another participant explained:

*“I hear that if you get HIV you will use ARV and nobody will know you have HIV, but with pregnancy everyone will know.” (FGD 4)*

There was consensus among participants that because pregnancy was not as discreetly manageable as HIV or other STIs, young women would be more likely to take preventive measures that would reduce their pregnancy risk more readily than preventive measures to reduce HIV or other STI risk.

### Theme 2: Although AGYW were aware of contraceptive methods, they expressed concerns and misconceptions about contraceptive use

When asked to detail their awareness regarding different kinds of contraceptives, participants felt that they and their peers were well-informed, with one participant in an FGD stating that:

*“Most girls are informed in this era so it is all about ignorance. If you ignore the information definitely you will face the consequences, so most girls are informed. It is just ignored.” (FGD 4)*

Another participant in the same group elaborated:

*“I guess right now I don’t think there is a girl who doesn’t know all the ways of preventing pregnancies and STIs. I really concur with her that it is just ignorance. Even if you ask today a girl who has just finished with her high school on ways of preventing HIV and STIs, they will tell you all of them.” (FGD 4)*

AGYWs were able to list and describe most major types of contraceptives, including injectables, EC pills, daily oral contraceptives, implants, condoms, and intrauterine devices. Many were able to detail important side effects and considerations, with one FGD participant stating:

*“When using Depo-Provera [injectable contraceptive], a person gets side effects like having heavy menses, or suppressed menses.” (FGD 05)*

Another interviewee noted that:

*“From my observation many people prefer injections because it is easier to forget taking the daily pills because sometimes…let us say that you are on a journey and forgot the pill…” (IDI, 20)*

However, alongside this general awareness regarding contraceptives, there were also a number of misconceptions that emerged among participants, especially around long-acting reversible contraceptives (LARCs). One of the most commonly held misconceptions was that the use of contraceptives, particularly before someone has successfully carried a pregnancy to term, would lead to infertility. This sentiment was expressed in the majority of interviews, with many participants noting that this misconception was their reason for not using contraceptives, especially LARCs:

*“I didn’t want to take that medicine that they usually say people should take…I hear they are not good…they can prevent you afterwards [from having a] child.” (IDI 03)*

Others expressed similar sentiments about a variety of contraceptives, with many young women noting that:

*“Girls don’t like Jadelle [the implant], because that is spoiling them. If you have that [implant] and fail to get a child in future you will regret. However, it is not difficult to accept if you have already delivered.” (FGD 01)*

*“People say that the fertility is reduced. The fertility is reduced so many girls opt for P2 [the EC pill].” (IDI 16)*

This widespread misconception that early use of contraceptives might have some effect on fertility spanned across contraceptive types. Participants noted that these misconceptions influence decisions about contraceptive use among young women, with one interviewee noting that *“you get discouraged by the beliefs people have out there” (FGD 03),* and many participants expressed that the perceived risk of infertility impeded many from trusting LARCs, injectables, and oral contraceptive pills as safe forms of pregnancy prevention. They also described beliefs regarding the negative impact of hormonal and long-term contraceptives on reproductive organs, cancer, and miscarriage, all of which deterred AGYW from using LARCs and other long-term hormonal contraceptives.

### Theme 3: EC pills were described as the preferred contraceptive choice among AGYW compared to longer-acting, more reliable contraceptive methods

Among our cohort, EC pills emerged as the most commonly discussed and popular form of contraception. As illustrated in prior themes, AGYW describe being motivated by a fear of pregnancy, yet, despite their adequate knowledge of contraceptive methods, also describe being reluctant to use contraception. When examining the actual contraceptives used by AGYW, they report that condoms, oral contraceptive pills, and other long-term and hormonal forms of birth control were avoided by their peers, reflecting that:

*“Not many people use condoms. The only thing they fear is getting pregnant but not HIV, so I do not think that the condoms work.” (FGD 01)*

*“You will get that…most girls in campus do not like having protected sex so you get that today they take P2 [the emergency pill], so they don’t care about themselves as long as I don’t get pregnant…the rest they don’t care.” (FGD 2)*

“P2” (or Postinor 2), the EC pill, was discussed by participants as a popular choice of birth control, though they felt that their peers were often ‘misusing’ the EC pill by adopting it as a primary form of contraception. One participant noted that:

*“There is the emergency pill but…when you use it so often it will not work. It should…only be used when maybe it [sex] was an accident…but mostly nowadays they are misused.” (IDI 12)*

Easily accessible at pharmacies, the EC pill offers adolescent girls a convenient, low-cost way to access contraceptives, thereby minimizing the need to make return visits (to collect pills) or to schedule an appointment in a public hospital (for an IUD, implant, or Depo Provera injection) in order to access pregnancy prevention. Participants also perceived the risk of side effects (including misconceived notions of infertility) to be lower with EC pills than with LARCs, making the EC pill the contraceptive method that they felt had the least consequences with use.

## Discussion

Our results demonstrate that AGYW who have recently become sexually active are aware of contraceptive method options for preventing pregnancy. However, AGYW also describe reluctance to use these contraceptive methods due to misconceptions about infertility and lack of guidance around side effects, and expressed the most concern about effects of LARCs. Despite these attitudes, AGYW expressed a strong “fear” of pregnancy, rooted in the physical visibility and associated shame of carrying a pregnancy, alongside the responsibilities and life changes that are associated with raising a child. Participants were knowledgeable about HIV risk and prevention, but often did not use condoms to mitigate this risk. Participants reported that AGYW were more likely to use contraceptive methods than to utilize condoms, due to greater concern for pregnancy as a negative outcome.

Previous research in Kenya and other regions of sub-Saharan Africa has shown that misconceptions about contraceptives are a major barrier to uptake and continuous use of contraceptives among adolescents, and that peers, communities, and partners are crucial in the development of these misconceptions [9, 21–23]. Our work builds on this body of research, but adds additional nuance, since we focused closely on the period of time around initiation of sexual activity and since almost all participants in our research were very clear that they did not yet want to become pregnant. Our research found that AGYW were confronted with difficult choices: wanting to avoid a pregnancy, but not finding acceptable methods to use based on social beliefs, leading to EC pill use or no use of contraceptives at all after unprotected sex.

AGYW in our study acknowledged that continuous contraceptive use was a more reliable way of achieving their goal of preventing unintended pregnancy. However, in FGDs they noted that their peers continued to rely mainly on EC pills, based on the belief that EC pills offered lower risk of side effects and better return to fertility. For AGYW who desire to prevent unintended pregnancy, the EC pill represents ease, convenience, and a perception of being a “safer alternative” with fewer adverse effects than more continuous use of contraception. These findings align with existing literature showing that EC pills are widely accepted by adolescents due to its ease of access and on-demand profile [14, 15]. Further, EC pill use does not require proactive planning and decision-making, which are necessary components of the use of long-term contraceptives.

Planning to use longer-acting contraception requires adolescents to peremptorily overcome long-standing societal shame and stigma and cognitive biases around contraceptive use [14, 24]. Other research from this cohort showed that first sexual intercourse was often unplanned and that a discourse around “decisions” about contraception and HIV prevention was not cognizant of the true nature of unexpected and unanticipated sexual activity [19]. This further demonstrates that after their first sexual activity, AGYW face challenges, including beliefs and stigma regarding contraceptive use, which result in utilization of EC pills as the most likely outcome. By recognizing these reasons for the appeal of EC pills, we can attempt to structure more effective interventions targeting behavioral change to promote the use of longer-term contraception among AGYW who want to prevent unplanned pregnancy. For example, counseling techniques should concentrate on enhancing awareness of EC use, alongside the need to consider alternate long-term contraceptive methods given that overuse of EC use may have minimal impact on unwanted pregnancy or abortion among individuals who engage in unprotected sexual intercourse [14, 24].

Other research has demonstrated strong cognitive dissonance among adolescents as they engage with the idea of long-term contraception use; namely that AGYW consider consistent use of long-acting or hormonal contraception as an ‘adult’ behavior and find it incongruous with their own self-perception as young people [24]. EC pills, therefore, represent a compromise, and allow AGYW to prevent unintended pregnancy without adopting the new, uncomfortable role of a contraceptive user. Rebranding long-acting contraceptives, with consideration of this cognitive dissonance, may lead to increased uptake among AGYW by helping to overcome the perception that regular use of contraception is solely for older women. Ultimately, future interventions to help “bridge” AGYW from EC pill use to more reliable contraceptives should employ multifaceted approaches that target adolescent attitudes and intentionality, but also tangibly reduce cognitive and structural barriers that dissuade AGYW from accessing contraceptives prior to engaging in sexual activity.

Our study has important limitations to note. First, since our sample was drawn from a single clinic in Thika, Kenya, it is limited in its geographical and population generalizability. All study participants were part of a reproductive health research cohort, and therefore had already decided to engage in an intervention for sexual and reproductive health. This did, however, have the benefit of allowing us to explore contraceptive and HIV prevention attitudes in an environment where information and access were not the main barriers to contraceptive use. Participants had received counseling about certain aspects of sexual and reproductive health and there may have been social desirability bias to endorse reproductive health practices that were part of this counseling.

## Conclusion

Understanding decision-making around contraceptive choice by AGYW is crucial to developing interventions and systems that can effectively override barriers to contraceptive uptake and reduce unplanned teenage pregnancy. Our qualitative work with AGYW in a youth friendly clinic showed that despite a clear preference for avoiding pregnancy, and knowledge and access to a variety of contraceptive options, use of LARCs remained low and EC pills were described as the most widely used contraceptive. Further, misinformation around adverse effects of contraceptive methods remains a challenge. It is clear that AGYW have a preference for on-demand methods that do not require planning in advance and have the lowest perceived side effect profile. AGYW preferences observed in this study are consistent with neuroscience observations about adolescent brains which typically prioritize peer input and short-term thinking over long term planning [25]. This work shows that novel contraceptive methods that are safe, reliable and can be used on-demand will continue to be preferred and popular among AGYW.

## Data Availability

This article does not provide public access to the data described, but the corresponding author may provide it upon reasonable request at kngure@pipsthika.org/ kngure@jkuat.ac.ke.

## Abbreviations

AGYW: Adolescent girls and young women
GHS: Girls health study
IDIs: In-depth interviews
FGDs: Focus group discussions
STI/HIV: Sexual transmitted infections/ human immunodeficiency virus
LARCs: Long-acting reversible contraceptives
EC pill: Emergency contraceptive pill
